# Benzyl­ammonium hexa­noate

**DOI:** 10.1107/S1600536812039931

**Published:** 2012-09-26

**Authors:** Mary H. Wood, Stuart M. Clarke

**Affiliations:** a BP Institute and Department of Chemistry, University of Cambridge, Cambridge, England

## Abstract

A binary mixture of benzyl­amine and hexa­noic acid has been reacted to form the title salt, C_7_H_10_N^+^·C_6_H_11_O_2_^−^. This crystal has a 1:1 stoichiometry of acid- and amine-derived species which contrasts with other related species which can have a number of other integer ratios of acid and amine components. The diffraction data indicate complete transfer of a proton from the acid to the amine to give the salt, comprising a cation and anion combination, with the formation of three hydrogen bonds around each ammonium group. This contrasts with other related species.

## Related literature

For spectroscopic studies of acid–amine complexes, see: Karlsson *et al.* (2000 ▶); Paivarinta *et al.* (2000 ▶); Kohler *et al.* (1981 ▶); Smith *et al.* (2001 ▶, 2002 ▶). For recent diffraction studies of acid–amine complexes, see: Jefferson *et al.* (2011 ▶); Sun *et al.* (2011 ▶).
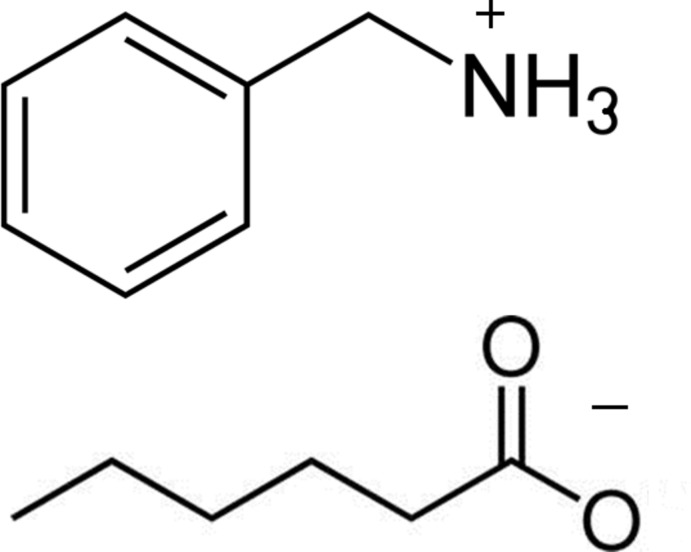


## Experimental

### 

#### Crystal data

C_7_H_10_N^+^·C_6_H_11_O_2_^−^
*M* *_r_* = 223.31
Triclinic, 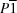

*a* = 5.7730 (3) Å
*b* = 7.7465 (4) Å
*c* = 15.1707 (8) Å
α = 98.318 (3)°
β = 90.638 (3)°
γ = 105.641 (2)°
*V* = 645.55 (6) Å^3^
*Z* = 2
Mo *K*α radiation
μ = 0.08 mm^−1^
*T* = 180 K
0.37 × 0.25 × 0.02 mm

#### Data collection

Nonius Kappa CCD diffractometer
Absorption correction: multi-scan (*SORTAV*; Blessing, 1995 ▶) *T*_min_ = 0.824, *T*_max_ = 1.000
9587 measured reflections
2915 independent reflections
1930 reflections with *I* > 2σ(*I*)
*R*_int_ = 0.060

#### Refinement

*R*[*F* ^2^ > 2σ(*F* ^2^)] = 0.068
*wR*(*F* ^2^) = 0.177
*S* = 1.04
2915 reflections
147 parameters
H-atom parameters constrained
Δρ_max_ = 0.59 e Å^−3^
Δρ_min_ = −0.33 e Å^−3^

### 

Data collection: *COLLECT* (Nonius, 1998 ▶); cell refinement: *SCALEPACK* (Otwinowski & Minor, 1997 ▶); data reduction: *DENZO* (Otwinowski & Minor, 1997 ▶) and *SCALEPACK*; program(s) used to solve structure: *SIR92* (Altomare *et al.*, 1994 ▶); program(s) used to refine structure: *SHELXL97* (Sheldrick, 2008 ▶); molecular graphics: *SHELXTL* (Sheldrick, 2008 ▶); software used to prepare material for publication: *SHELXL97*.

## Supplementary Material

Crystal structure: contains datablock(s) I, global. DOI: 10.1107/S1600536812039931/mw2086sup1.cif

Structure factors: contains datablock(s) I. DOI: 10.1107/S1600536812039931/mw2086Isup2.hkl

Supplementary material file. DOI: 10.1107/S1600536812039931/mw2086Isup3.cml

Additional supplementary materials:  crystallographic information; 3D view; checkCIF report

## Figures and Tables

**Table 1:** Hydrogen-bond geometry (Å, °)

*D*—H⋯*A*	*D*—H	H⋯*A*	*D*⋯*A*	*D*—H⋯*A*
N1—H1*A*⋯O1	0.91	1.99	2.890 (3)	169
N1—H1*B*⋯O2^i^	0.91	1.81	2.705 (3)	169
N1—H1*C*⋯O1^ii^	0.91	1.88	2.769 (3)	164
C1—H1*D*⋯O2^iii^	0.99	2.45	3.366 (3)	154
C7—H7⋯N1	0.95	2.58	2.902 (3)	100
C7—H7⋯O1^ii^	0.95	2.53	3.347 (3)	144

Symmetry codes: (i) 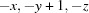; (ii) 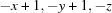; (iii) 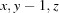.

**Fig. 1.**
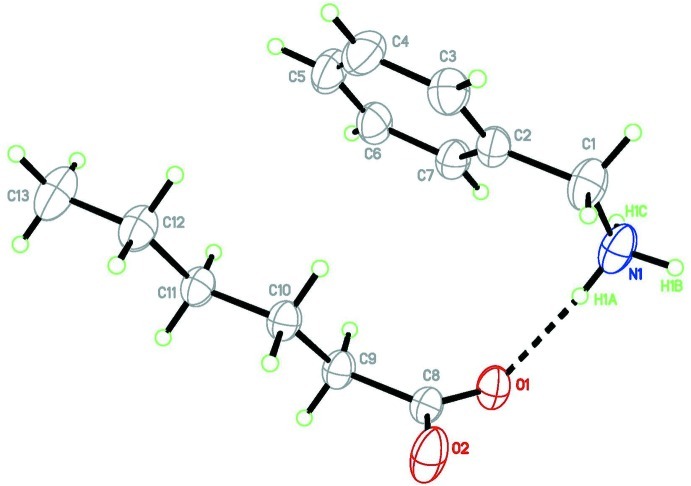
Perspective view of the asymmetric unit showing one of the three N—H···O hydrogen bonds

**Fig. 2.**
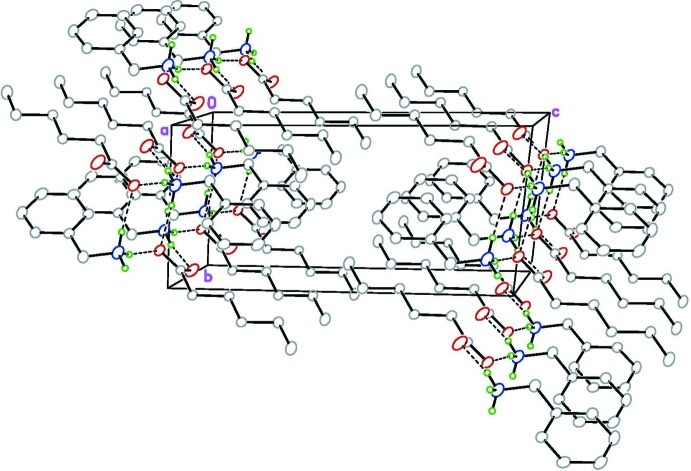
Illustration of the packing. Hydrogen bonds are shown by dashed lines.

## supplementary crystallographic information

### Comment

Several studies, mainly spectroscopy-based, have reported the existence of stable complexes formed between simple fatty acids and amines, both alkyl and aromatic-based *e.g.* (Karlsson *et al.*, 2000). Numerous 1:1 acid:amine complexes have been identified; in addition, various examples of 2:1 and 3:1 adducts have been discovered, usually in an acid-rich environment (Sun *et al.*, 2011; Kohler *et al.*, 1981). Interestingly, no amine-rich complexes have yet been observed; indeed, it has been proposed that these would be highly unstable were they to form (Paivarinta *et al.*, 2000), although there is a report of a diamine complex formed between methylamine and dnsa (3, 5-dinitrosalicyclic acid) due to deprotonation of the phenolic group in the acid (Smith *et al.*, 2001; Smith *et al.*, 2002).

The 1:1 acid:amine complexes are generally considered to derive their stablity from the complete transfer of a proton from the acid to the amine with subsequent cation-anion electrostatic interaction and strong hydrogen-bond formation. In 2:1 or high stoichiometry complexes, the hydrogen bond is considered to extend over the three (or more) species involved.

The 1:1 complex of hexanoic acid and benzylamine forms by reaction of the two species with complete proton transfer from the acid to the base. Each ammonium ion in this salt can now form three hydrogen bonds, one of which is shown in Fig. 1 and all three in Fig. 2. This work follows from similar findings reported by (Jefferson *et al.*, 2011) who report the structure of a 1:1 complex of octanoic acid and decylamine using the same experimental method of preparation. This work differs from the previous study concerning complex formation with an aromatic amine, rather than an alkyl amine reported previously. In general, few examples of such single-crystal data exist for such complexes, due mainly to the difficulty of growing suitable crystals. The molecular arrangement of the alkyl and aromatic groups is also somewhat surprising. One might have imagined the aromatic rings interacting strongly together and 'stacking' separately from the alkyl chains of the hexanoic acid. However, they appear to be arranged adjacent to each other in the 1:1 crystal, with the planes of the aromatic ring and the alkyl chain backbone essentially parallel, Fig. 2.

### Experimental

Hexanoic acid and benzylamine, with purities of 99.5% and 99.7% respectively as determined by titration and GC, were purchased from Sigma Aldrich and used without further purification. The crystals were grown by pipetting a small volume (approximately 1 ml) of each into two small vials, and leaving both within a larger vial over a number of weeks all under an inert atmosphere of nitrogen. After this period numerous crystals were observed, with particularly abundant growth on a polypropylene surface that had been left therein as a nucleating surface. The inert atmosphere was employed to minimize reaction of the amine with atmospheric CO_2_, which can make such complexation studies difficult (Sun *et al.* 2011).

Elemental analysis gave values of 69.85%, 6.22%, 9.42% and 14.52% for carbon, nitrogen, hydrogen and oxygen respectively. For a 1:1 complex these values are expected to be 69.92%, 6.27%, 9.48% and 14.32%, in excellent agreement. The 1:1 stoichiometry also agrees with the crystal structure determination given here. The experimental sample temperature 180 K represents a compromise of several factors. It is selected as the temperature which is cold enough to get improved thermal factors but not so cold that the crystals fracture and it is a temperature at which the cryostream can run efficiently for an extended period.

### Crystal data

**Table:**

C_7_H_10_N^+^·C_6_H_11_O_2_^−^	*Z* = 2
*M**_r_* = 223.31	*F*(000) = 244
Triclinic, *P*1	*D*_x_ = 1.149 Mg m^−^^3^
Hall symbol: -P 1	Mo *K*α radiation, λ = 0.71073 Å
*a* = 5.7730 (3) Å	Cell parameters from 18567 reflections
*b* = 7.7465 (4) Å	θ = 1.0–27.5°
*c* = 15.1707 (8) Å	µ = 0.08 mm^−^^1^
α = 98.318 (3)°	*T* = 180 K
β = 90.638 (3)°	Block, colourless
γ = 105.641 (2)°	0.37 × 0.25 × 0.02 mm
*V* = 645.55 (6) Å^3^	

### Data collection

**Table:**

Nonius Kappa CCD diffractometer	1930 reflections with *I* > 2σ(*I*)
Radiation source: fine-focus sealed tube	*R*_int_ = 0.060
Thin slice ω and φ scans	θ_max_ = 27.5°, θ_min_ = 3.6°
Absorption correction: multi-scan (*SORTAV*; Blessing, 1995)	*h* = −7→7
*T*_min_ = 0.824, *T*_max_ = 1.000	*k* = −10→10
9587 measured reflections	*l* = −19→19
2915 independent reflections	

### Refinement

**Table:**

Refinement on *F*^2^	Primary atom site location: structure-invariant direct methods
Least-squares matrix: full	Secondary atom site location: difference Fourier map
*R*[*F*^2^ > 2σ(*F*^2^)] = 0.068	Hydrogen site location: inferred from neighbouring sites
*wR*(*F*^2^) = 0.177	H-atom parameters constrained
*S* = 1.04	*w* = 1/[σ^2^(*F*_o_^2^) + (0.0564*P*)^2^ + 0.4841*P*] where *P* = (*F*_o_^2^ + 2*F*_c_^2^)/3
2915 reflections	(Δ/σ)_max_ < 0.001
147 parameters	Δρ_max_ = 0.59 e Å^−^^3^
0 restraints	Δρ_min_ = −0.33 e Å^−^^3^

### Special details

**Table:**

Experimental. The data is moderately weak at high angle (66% observed), a fact reflected in the rather large K value in the analysis of variance.Absorption correction: multi-scan from symmetry-related measurements Sortav (Blessing, 1995)
Geometry. All e.s.d.'s (except the e.s.d. in the dihedral angle between two l.s. planes) are estimated using the full covariance matrix. The cell e.s.d.'s are taken into account individually in the estimation of e.s.d.'s in distances, angles and torsion angles; correlations between e.s.d.'s in cell parameters are only used when they are defined by crystal symmetry. An approximate (isotropic) treatment of cell e.s.d.'s is used for estimating e.s.d.'s involving l.s. planes.
Refinement. Refinement of *F*^2^ against ALL reflections. The weighted *R*-factor *wR* and goodness of fit *S* are based on *F*^2^, conventional *R*-factors *R* are based on *F*, with *F* set to zero for negative *F*^2^. The threshold expression of *F*^2^ > σ(*F*^2^) is used only for calculating *R*-factors(gt) *etc*. and is not relevant to the choice of reflections for refinement. *R*-factors based on *F*^2^ are statistically about twice as large as those based on *F*, and *R*- factors based on ALL data will be even larger.

### Fractional atomic coordinates and isotropic or equivalent isotropic displacement parameters (Å^2^)

**Table:**

	*x*	*y*	*z*	*U*_iso_*/*U*_eq_	
N1	0.2211 (3)	0.3348 (3)	0.04302 (13)	0.0433 (5)	
H1A	0.2402	0.4524	0.0359	0.052*	
H1B	0.0980	0.2617	0.0056	0.052*	
H1C	0.3594	0.3036	0.0301	0.052*	
O1	0.3458 (3)	0.7110 (2)	0.01788 (10)	0.0369 (4)	
O2	0.1113 (3)	0.8658 (2)	0.08527 (12)	0.0499 (5)	
C1	0.1663 (4)	0.3134 (4)	0.13431 (15)	0.0435 (6)	
H1D	0.1370	0.1838	0.1402	0.052*	
H1E	0.0153	0.3472	0.1473	0.052*	
C2	0.3600 (4)	0.4245 (3)	0.20347 (14)	0.0330 (5)	
C3	0.3043 (4)	0.4336 (3)	0.29247 (16)	0.0406 (6)	
H3	0.1468	0.3744	0.3079	0.049*	
C4	0.4766 (5)	0.5283 (4)	0.35889 (16)	0.0477 (7)	
H4	0.4366	0.5337	0.4196	0.057*	
C5	0.7059 (5)	0.6149 (4)	0.33769 (17)	0.0476 (7)	
H5	0.8237	0.6795	0.3836	0.057*	
C6	0.7630 (4)	0.6073 (3)	0.24995 (17)	0.0403 (6)	
H6	0.9204	0.6679	0.2350	0.048*	
C7	0.5917 (4)	0.5113 (3)	0.18271 (15)	0.0353 (5)	
H7	0.6335	0.5052	0.1222	0.042*	
C8	0.3117 (4)	0.8349 (3)	0.07608 (14)	0.0295 (5)	
C9	0.5207 (4)	0.9530 (3)	0.13816 (14)	0.0317 (5)	
H9A	0.5665	1.0766	0.1220	0.038*	
H9B	0.6610	0.9029	0.1296	0.038*	
C10	0.4613 (4)	0.9650 (3)	0.23628 (14)	0.0318 (5)	
H10A	0.3210	1.0151	0.2450	0.038*	
H10B	0.4160	0.8416	0.2527	0.038*	
C11	0.6721 (4)	1.0840 (3)	0.29756 (14)	0.0335 (5)	
H11A	0.7103	1.2091	0.2834	0.040*	
H11B	0.8151	1.0385	0.2856	0.040*	
C12	0.6235 (4)	1.0890 (3)	0.39634 (15)	0.0410 (6)	
H12A	0.4842	1.1386	0.4088	0.049*	
H12B	0.5800	0.9635	0.4102	0.049*	
C13	0.8384 (5)	1.2031 (4)	0.45713 (16)	0.0548 (7)	
H13A	0.7975	1.2008	0.5195	0.082*	
H13B	0.8795	1.3285	0.4451	0.082*	
H13C	0.9765	1.1536	0.4459	0.082*	

### Atomic displacement parameters (Å^2^)

**Table:**

	*U*^11^	*U*^22^	*U*^33^	*U*^12^	*U*^13^	*U*^23^
N1	0.0280 (10)	0.0569 (13)	0.0401 (11)	0.0120 (9)	−0.0066 (8)	−0.0083 (10)
O1	0.0315 (8)	0.0390 (9)	0.0364 (9)	0.0099 (7)	−0.0016 (7)	−0.0069 (7)
O2	0.0281 (9)	0.0604 (11)	0.0541 (11)	0.0168 (8)	−0.0111 (7)	−0.0223 (9)
C1	0.0300 (12)	0.0537 (15)	0.0380 (13)	0.0004 (11)	0.0007 (10)	−0.0003 (11)
C2	0.0305 (11)	0.0324 (12)	0.0336 (12)	0.0080 (9)	−0.0025 (9)	−0.0011 (9)
C3	0.0390 (13)	0.0409 (13)	0.0392 (13)	0.0082 (10)	0.0040 (10)	0.0030 (10)
C4	0.0592 (17)	0.0549 (16)	0.0300 (12)	0.0199 (13)	−0.0033 (12)	0.0019 (11)
C5	0.0488 (16)	0.0467 (15)	0.0431 (14)	0.0134 (12)	−0.0185 (12)	−0.0063 (11)
C6	0.0310 (12)	0.0369 (13)	0.0487 (14)	0.0056 (10)	−0.0071 (10)	0.0006 (11)
C7	0.0300 (12)	0.0365 (12)	0.0365 (12)	0.0067 (9)	−0.0026 (9)	0.0014 (10)
C8	0.0260 (11)	0.0302 (11)	0.0297 (11)	0.0048 (9)	−0.0014 (8)	0.0024 (9)
C9	0.0249 (11)	0.0343 (12)	0.0323 (11)	0.0050 (9)	−0.0017 (9)	−0.0003 (9)
C10	0.0268 (11)	0.0329 (12)	0.0324 (11)	0.0046 (9)	−0.0017 (9)	0.0009 (9)
C11	0.0301 (11)	0.0341 (12)	0.0318 (11)	0.0035 (9)	−0.0029 (9)	0.0013 (9)
C12	0.0392 (13)	0.0440 (14)	0.0332 (12)	0.0027 (11)	−0.0027 (10)	0.0016 (10)
C13	0.0500 (16)	0.0684 (19)	0.0341 (13)	0.0016 (14)	−0.0057 (12)	−0.0016 (13)

### Geometric parameters (Å, º)

**Table:**

N1—C1	1.447 (3)	C6—H6	0.9500
N1—H1A	0.9100	C7—H7	0.9500
N1—H1B	0.9100	C8—C9	1.520 (3)
N1—H1C	0.9100	C9—C10	1.527 (3)
O1—C8	1.265 (3)	C9—H9A	0.9900
O2—C8	1.248 (3)	C9—H9B	0.9900
C1—C2	1.511 (3)	C10—C11	1.522 (3)
C1—H1D	0.9900	C10—H10A	0.9900
C1—H1E	0.9900	C10—H10B	0.9900
C2—C3	1.388 (3)	C11—C12	1.525 (3)
C2—C7	1.388 (3)	C11—H11A	0.9900
C3—C4	1.383 (3)	C11—H11B	0.9900
C3—H3	0.9500	C12—C13	1.522 (3)
C4—C5	1.378 (4)	C12—H12A	0.9900
C4—H4	0.9500	C12—H12B	0.9900
C5—C6	1.372 (4)	C13—H13A	0.9800
C5—H5	0.9500	C13—H13B	0.9800
C6—C7	1.390 (3)	C13—H13C	0.9800
			
C1—N1—H1A	109.5	O1—C8—C9	119.66 (18)
C1—N1—H1B	109.5	C8—C9—C10	112.88 (17)
H1A—N1—H1B	109.5	C8—C9—H9A	109.0
C1—N1—H1C	109.5	C10—C9—H9A	109.0
H1A—N1—H1C	109.5	C8—C9—H9B	109.0
H1B—N1—H1C	109.5	C10—C9—H9B	109.0
N1—C1—C2	114.89 (19)	H9A—C9—H9B	107.8
N1—C1—H1D	108.5	C11—C10—C9	112.24 (18)
C2—C1—H1D	108.5	C11—C10—H10A	109.2
N1—C1—H1E	108.5	C9—C10—H10A	109.2
C2—C1—H1E	108.5	C11—C10—H10B	109.2
H1D—C1—H1E	107.5	C9—C10—H10B	109.2
C3—C2—C7	118.7 (2)	H10A—C10—H10B	107.9
C3—C2—C1	117.8 (2)	C10—C11—C12	113.42 (18)
C7—C2—C1	123.4 (2)	C10—C11—H11A	108.9
C4—C3—C2	120.4 (2)	C12—C11—H11A	108.9
C4—C3—H3	119.8	C10—C11—H11B	108.9
C2—C3—H3	119.8	C12—C11—H11B	108.9
C5—C4—C3	120.5 (2)	H11A—C11—H11B	107.7
C5—C4—H4	119.7	C13—C12—C11	113.0 (2)
C3—C4—H4	119.7	C13—C12—H12A	109.0
C6—C5—C4	119.6 (2)	C11—C12—H12A	109.0
C6—C5—H5	120.2	C13—C12—H12B	109.0
C4—C5—H5	120.2	C11—C12—H12B	109.0
C5—C6—C7	120.3 (2)	H12A—C12—H12B	107.8
C5—C6—H6	119.8	C12—C13—H13A	109.5
C7—C6—H6	119.8	C12—C13—H13B	109.5
C2—C7—C6	120.4 (2)	H13A—C13—H13B	109.5
C2—C7—H7	119.8	C12—C13—H13C	109.5
C6—C7—H7	119.8	H13A—C13—H13C	109.5
O2—C8—O1	122.74 (19)	H13B—C13—H13C	109.5
O2—C8—C9	117.60 (19)		

### Hydrogen-bond geometry (Å, º)

**Table:**

*D*—H···*A*	*D*—H	H···*A*	*D*···*A*	*D*—H···*A*
N1—H1*A*···O1	0.91	1.99	2.890 (3)	169
N1—H1*B*···O2^i^	0.91	1.81	2.705 (3)	169
N1—H1*C*···O1^ii^	0.91	1.88	2.769 (3)	164
C1—H1*D*···O2^iii^	0.99	2.45	3.366 (3)	154
C7—H7···N1	0.95	2.58	2.902 (3)	100
C7—H7···O1^ii^	0.95	2.53	3.347 (3)	144

Symmetry codes: (i) −*x*, −*y*+1, −*z*; (ii) −*x*+1, −*y*+1, −*z*; (iii) *x*, *y*−1, *z*.
